# Developments and Assessments of Crude Tea Saponin-Incorporated Silica Nanoparticles for Their Bioactivity Improvement

**DOI:** 10.3390/jfb16100390

**Published:** 2025-10-17

**Authors:** Tanrada Likitsatian, Pimpisid Koonyosying, Sittiruk Roytrakul, Patcharawan Srisilapanan, Somdet Srichairatanakool, Jetsada Ruangsuriya

**Affiliations:** 1 Department of Biochemistry, Faculty of Medicine, Chiang Mai University, Chiang Mai 50200, Thailand; tanrada.likit@gmail.com (T.L.); pimpisid.k@cmu.ac.th (P.K.); somdet.s@cmu.ac.th (S.S.); 2 National Center for Genetic Engineering and Biotechnology (BIOTEC), National Science and Technology Development Agency, Pathum Thani 12120, Thailand; sittiruk@biotec.or.th; 3 School of Dentistry, Phayao University, Phayao 56000, Thailand; patcharawan.sr@up.ac.th; 4 Functional Food Research Unit, Multidisciplinary Research Institute, Chiang Mai University, Chiang Mai 50200, Thailand

**Keywords:** crude tea saponin, silica nanoparticles, bioavailability enhancement, cytotoxicity reduction, oxidative stress

## Abstract

The use of saponins with biosurfactant, antioxidant, anti-inflammatory, and anti-cancer properties is limited by their toxicity and bioavailability. This study focused on the fabrication, characterization, and bioactivity of crude tea saponin (TS) and TS-incorporated silica nanoparticles (TSNPs). Our results showed that TS contained seven saponins and that TSNPs had an average diameter of 200–300 nm, a negative surface charge, and high polydispersity. Fourier Transform Infrared Spectroscopy (FTIR) revealed an incorporation bond of Si-O- and -OH controlling releasing behavior with t_50_ = 24 h. Using HaCaT cells, it was demonstrated that TSNPs reduced cytotoxicity. Reactive oxygen species (ROS) production was lowered in both TS and TSNP treatments, with significantly greater efficacy at higher concentrations. Additionally, TSNPs significantly accelerated cell migration in the wound closure model as efficiently as TGFβ. Together, these findings offer promising TSNPs for biomedical applications and therapeutic agents due to their antioxidant properties, cytotoxicity protection, and wound closure acceleration.

## 1. Introduction

Saponins are widely distributed in many plants such as soybeans (*Glycine max*), yucca (*Yucca schidigera*), quinoa (*Chenopodium quinoa*), and green tea (*Camellia oleifera*). While soybeans and quinoa are widely acknowledged as being rich sources of saponins, their primary use as food crops limits the feasibility of large-scale saponin extraction due to potential conflicts with food supply chains. Similarly, yucca contains saponins with potent surfactant properties; however, its fibrous composition makes extraction less efficient and requires more complex and energy-intensive methods. In contrast, green tea provides a sustainable and abundant source of saponins with several advantages over other plants. Green tea has been widely cultivated for the polyphenols and saponins it contains, along with the basic tea production, with those compounds being extracted from tea residues after tea production. The simple aqueous or ethanol-based methods used for saponin extraction from green tea are highly efficient and are easily reproducible, making it a cost-effective and environmentally friendly option [1,2]. Green tea saponins from *Camellia oleifera* also exhibit great bioactive effects with potent antioxidant and anti-inflammatory properties, which are particularly beneficial for biomedical applications [3,4]. However, their poor bioavailability and high cytotoxicity to skin cells limit their therapeutic applications [5,6,7]. The incorporation of saponins into nanocarriers, particularly in the case of silica nanoparticles (SNPs), offers a promising approach due to the high biocompatibility, stability, and encapsulation potential of these SNPs [8,9,10].

Nanosilica has been widely utilized to improve the mechanical, structural, and restorative properties of dental materials. For example, nanosilica enhances the mechanical strength and wear resistance of polymethyl methacrylate (PMMA) denture bases [8], assisting in the remineralization of erosive dental lesions [11] and improving the microhardness and setting time of glass-ionomer cement [12]. These applications demonstrate the versatility of nanosilica in biomedical applications, suggesting its potential as a delivery vehicle for saponins to improve therapeutic outcomes [13,14]. Silica nanoparticles (SNPs) are ideal carriers for tea saponins (TS) due to their stability, large surface area, and controlled release capabilities [13,14]. The saponins from *Camellia oleifera,* including Camelliasaponin A2, Camelliasaponin B2, Assamsaponin E, and Matesaponin 4, are particularly promising saponins because of their potent antioxidant properties [8,11,12]. Previous studies have shown that combining saponins with silica nanoparticles could enhance cellular response and therapeutic efficacy [7,9,15,16]. The development of TSNPs with a novel approach to optimize the delivery and safety profile of saponins for biomedical applications is required.

Saponins have been encapsulated in nanosilica systems to enhance bioavailability and therapeutic potential. Saponin–cholesterol nanostructures for drug delivery have been created to improve the stability and bioavailability of saponins [14,16]. Similarly, incorporation of saponin micelles into silica networks for functional applications has been developed to improve intestinal adsorption [9]. In addition, saponin-based foam detergents stabilized by silica nanoparticles have demonstrated stability and enhanced activity of the bioactive compounds [15]. These findings support the efficacy of silica nanoparticles in improving the delivery and functionality of bioactive saponins.

The aim of this study was to develop crude tea saponin-incorporated silica nanoparticles (TSNPs) to enhance the bioavailability, efficacy, and safety of the saponins in the tea. The antioxidant, cytotoxicity, and cell migration properties of TSNPs were compared with the actions of crude tea saponins (TS) alone.

## 2. Materials and Methods

### 2.1. Plant Materials

Crude tea saponins (TS) from *Camellia oleifera* (*C. oleifera*) extracts were provided by Xi’an International Healthcare Factory Co., Ltd. (Xi’an, China).

### 2.2. Chemicals

Tetraethyl orthosilicate (TEOS, CAS No. 78-10-4), 3-(4,5-dimethylthiazol-2-yl)-2,5-diphenyl tetrazolium bromide (MTT), 2,2-diphenyl-1-picrylhydrazyl (DPPH), 2,2′-azino-bis(3-ethylbenzthiazoline-6-sulfonic acid) (ABTS), potassium persulfate, and 5-fluorouracil (5-FU, CAS No. 51-21-8, ≥99% purity) were purchased from Sigma-Aldrich (St. Louis, MO, USA). Ammonia solution, absolute ethanol, and formic acid (≥99%) were purchased from Merck (Darmstadt, Germany). Fetal bovine serum (FBS), Dulbecco’s Modified Eagle Medium (DMEM), penicillin/streptomycin solution, phosphate-buffered saline (PBS), and dimethyl sulfoxide (DMSO) were obtained from Gibco, Thermo Fisher Scientific (Waltham, MA, USA). In addition, QuEChERS dispersive SPE kit (Agilent Technologies, Santa Clara, CA, USA) for fat and pigment removal, 2′,7′-Dichlorodihydrofluorescein diacetate (H2DCFDA) (Invitrogen Molecular Probes, Carlsbad, CA, USA), recombinant human transforming growth factor-β (TGF-β) and (PeproTech, Rocky Hill, NJ, USA) were also commercially provided for this study. Ultrapure water (18.2 MΩ·cm) from a Milli-Q water system (Millipore, Burlington, MA, USA) was used in all experiments.

### 2.3. Phytochemical Identification

The identification method employed LC-MS, which was run on QTOF-MS (100–1700 *m*/*z*). First, 100 μg/mL of samples was prepared in 0.01% formic acid in ethanol (1:1, *v*/*v*). Then, sample purification was performed with a QuEChERS dispersive SPE kit designed for fat and pigment removal (Agilent Technology, Santa Clara, CA, USA). The Agilent 1290 Infinity II series coupled to a 6546 LC/Q-TOF instrument from Agilent Tech., Santa Clara, CA, USA, was used, which included a degasser, binary pump, and column oven. Chromatographic separation was carried out using a ZORBAX Eclipse Plus C18 column (2.1 × 150 mm, 1.8 µm), mobile phases 50–100% acetonitrile; flow-rate at 0.2 mL/min; and mass spectrometry through electrospray ionization (ESI) performed in negative mode in accordance with the protocol described. Identification of compounds was achieved by consulting an online database [17].

### 2.4. Antioxidant Activity of Crude Tea Saponins (TS) of C. oleifera

The antioxidant activity of the aqueous, methanol, and crude saponins of *C*. *oleifera* (15.62 to 125 μg/mL) was evaluated using DPPH and ABTS assays.

#### 2.4.1. DPPH Assay

The DPPH radical scavenging assay was used to test the ability of TS to neutralize the DPPH radical, and the protocols are described elsewhere with minor modifications [18]. L-ascorbic acid was also used as a reference standard. Briefly, 20 µL of TS at various concentrations (15.62 to 125 μg/mL) was mixed with 180 µL of 0.1 mM DPPH solution in a 96-well plate. After 20 min of incubation in the dark, absorbance was measured at 520 nm. The percentage of inhibition was calculated using the method described in [19].

#### 2.4.2. ABTS Assay

The ABTS radical scavenging assay was used to test the ability of TS to neutralize a cationic radical of ABTS•^+^, and the protocols are described elsewhere with slight modifications [20]. The ABTS•^+^ cation radical was generated by allowing 7 mM ABTS to react with 4.9 mM potassium persulfate in equal volumes and incubating the mixture overnight in the dark. The ABTS•^+^ solution was diluted to an absorbance of 0.700–0.720 at 734 nm before use. Subsequently, 5 µL of TS at various concentrations (15.62 to 125 μg/mL) was added to 4 mL of ABTS•^+^ solution and incubated in the dark for 2 h. Absorbance was recorded at 734 nm. The percentage of scavenging was calculated using the method described in [19].

### 2.5. Fabrication of Crude Tea Saponin Incorporated with Silica Nanoparticles (TSNPs)

#### 2.5.1. Synthesis of Silica Nanoparticles (SNPs)

**Figure 1:**
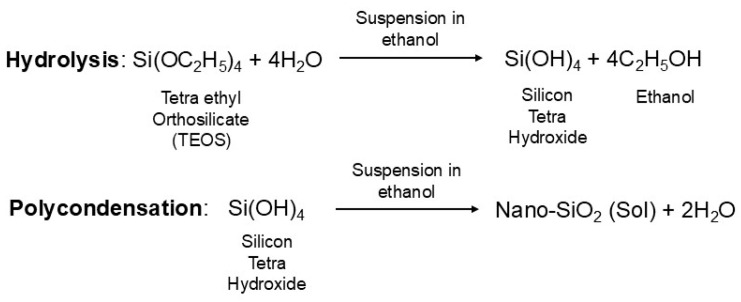
The sol–gel process for the synthesis of silica nanoparticles (SNPs) [21].

SNPs were synthesized using a sol–gel process based on two principal stages: hydrolysis and polycondensation (Figure 1).

First, 5 mL of TEOS (78-10-4, Sigma-Aldrich) was mixed with 30 mL of ethanol and stirred for 5 min. Then, sonication was performed for 10 min at 50 °C to ensure homogeneity. Next, 1 mL of deionized water (DI H2O) was added to the mixture under sonication, and the mixture was stirred for five minutes before the continuation of sonication for one and a half hours. Thereafter, 2 mL of ammonia was used as a catalyst to induce gelation, and the mixture was stirred for 10 min before sonication for 3 h. The sol–gel was then aged for 30 min before filtration with 20–25 μm filters following 8 μm filters. 

**Figure 2:**
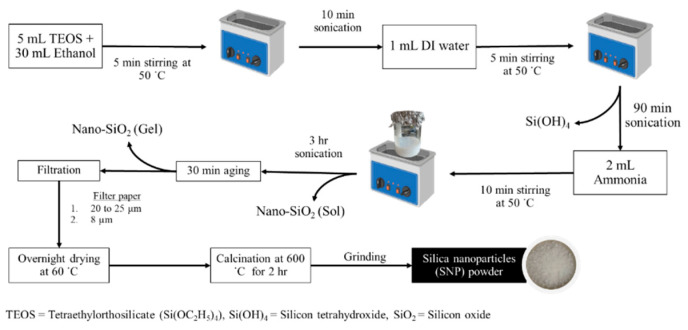
Fabrication diagram of the preparation of silica nanoparticles (SNPs) [21].

The ethanolic fraction was collected and dried with ambient air overnight. Finally, calcination was carried out at 600 °C for two hours, and the products were ground into SNP powder [21] (Figure 2).

#### 2.5.2. Preparation of Crude Saponin-Incorporated Silica Nanoparticles (TSNPs)

**Figure 3:**
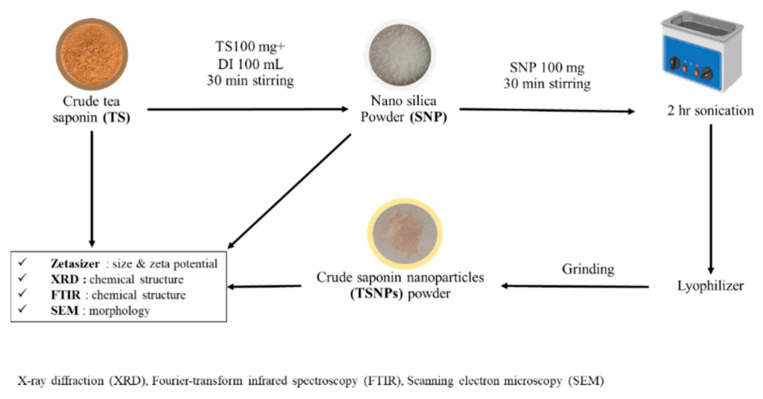
Fabrication diagram of crude saponin-incorporated silica nanoparticles (TSNPs). Before biological testing, physicochemical properties were characterized with Zetasizer for particle size and electrical potential, with X-ray diffraction (XRD) for chemical structure: particle crystallography, with Fourier transform infrared spectroscopy (FTIR) for chemical structure: surface chemistry, and with scanning electron microscopy (SEM) for surface morphology.

First, 100 mg of TS was dissolved in 100 mL of DI H2O and stirred for 30 min. Then, 100 mg SNPs were added, with the mixture being stirred for another 30 min. Subsequently, sonication was performed for two hours. Solid mixtures were obtained, and lyophilization was performed overnight. The dry products were ground using a mortar and pestle. Finally, TSNPs were fabricated from 100 mg of SNPs and 100 mg of TS (Figure 3).

### 2.6. Characterization of Crude Saponin-Incorporated Silica Nanoparticles (TSNPs)

#### 2.6.1. Scanning Electron Microscopy (SEM)

SEM was utilized to reveal the surface morphology of the samples. A slotted specimen stub (AGG301D Agar Scientific Ltd., Essex, UK), a 12.5 mm aluminum pin stub, was coated with some droplets of the sample suspension and allowed to air-dry under a light. After complete drying, the sample was gold-coated and observed with a field-emission scanning electron microscope (JSM-7600F, JEOL Ltd., Tokyo, Japan).

#### 2.6.2. Analysis of Zeta Size and Potentials

First, 100 μL of the sample was dissolved in 900 μL DI H2O and placed into a cuvette. The cuvette was inserted into the Malvern Zeta Instrument 3000 (Malvern Instrument, Worcestershire, UK), and the measurements were conducted at a temperature of 25 °C and a scattering angle of 90 °C. Material dispersion and refractive indices were calibrated to 1.365 and 1.330, respectively. The viscosity (CP) was set at 0.8872, while the dielectric constant was 78.5 [8,9].

#### 2.6.3. X-Ray Diffractometry (XRD) Analysis

Powder X-ray diffraction data were obtained using a Rigaku MiniFlex 600 powder X-ray diffractometer (Rigaku, Tokyo, Japan) with Cu Kα radiation (λ = 1.5406 Å) using a Ge monochromator. The measurements covered a Bragg angle 2θ range from 5° to 60° with 0.01° steps, utilizing a fast scanning mode to accommodate the air sensitivity of the samples. The diffraction patterns were analyzed via the LeBail method using Jana 2006.

#### 2.6.4. Fourier Transform Infrared Spectroscopy (FTIR)

The infrared absorption spectra of the powdered samples were recorded using FTIR. The FTIR spectra were obtained using a Nicolet iS 50 FT-IR Spectrometer (Thermo Fisher Scientific Inc., Waltham, MA, USA) in the spectral range of 4000-400 cm^−1^ with a resolution of 4 cm^−1^. Each spectrum was acquired by averaging 32 scans per sample. For sample preparation, the powdered samples were mixed with potassium bromide (KBr) in a 1:100 ratio and compressed into pellets under 10 MPa pressure for 2 min before measurement. This approach ensured optimal signal quality and minimized spectral noise.

### 2.7. High-Performance Liquid Chromatography (HPLC) Analysis of Crude Tea Saponin (TS)

HPLC was used to determine the presence of saponins in TS and the nanoparticles. The column was a 4 µm 4.6 × 250 mm Eclipse Plus C18 (Agilent, Santa Clara, CA, USA). First, 10 µL of TS or TSNPs dissolved in 80% ethanol were injected into the HPLC machine with UV detection (HPLC-UV) at 280 nm (1260 Infinity, Agilent, Santa Clara, CA, USA). The mobile phase was composed of DI H_2_O as Solvent A and methanol as Solvent B, mixed in the gradient elution program with the flow rate set at 0.5 mL/min. The chromatogram and the fingerprint were analyzed for saponin compounds. The peak area under the curve method was used along with the standard calibration curve obtained from different standard compounds for saponin quantification [2,22]. Both TS and TSNP samples were injected into the HPLC, identifying the saponin peaks based on the TS retention time and creating a saponin concentration curve using a series of dilutions. The concentration of the saponins in TSNPs was calculated by comparing their main peak area to the saponin concentration curve of dilutions. The limitation of this method was the lack of a standard saponin, which may affect the accuracy and reliability of the quantification. However, this study manipulated the saponin amount based on the established estimation to ensure consistency in the results [2].

### 2.8. In Vitro Release of Saponins from Crude Tea Saponin (TS) and Crude Saponin-Incorporated Silica Nanoparticles (TSNPs)

An in vitro drug release assay was performed to compare the release profiles of TS and crude tea saponin-loaded silica nanoparticles (TSNPs). Both formulations (TS at 25.0 mg/mL and TSNPs at 29.4 mg/mL in 10 mL of 80% ethanol) were loaded into dialysis bags with a molecular weight cut-off (MWCO) of 3.5 kDa (Shijiazhuang Zitong Import & Export Co., Ltd., Shijiazhuang, China). The bags were immersed in 20 mL of 80% ethanol in a 50 mL beaker and incubated under continuous magnetic stirring at room temperature. At each time point up to 48 h, 0.5 mL of the dissolution was collected into a vial for HPLC analysis [23]. Released tea saponins were quantified using HPLC, and the concentration at each time point was estimated using the method described in Section 2.5. The percentages of cumulative release were calculated and plotted into graphs [23,24]. The time required to release 50% (t50) and 90% (t90) of the drug was estimated. These parameters are commonly reported for comparative analysis of drug release profiles, especially in sustained delivery systems [25].

### 2.9. Biological Assays

#### 2.9.1. Cell and Cell Culture

Human keratinocyte cells (HaCaT cells), obtained from the American Type Culture Collection (ATCC, Manassas, VA, USA), were maintained in Dulbecco’s Modified Eagle’s Medium (DMEM) supplemented with 10% fetal bovine serum (FBS) and 1% penicillin/streptomycin, known as the complete medium. The cells were cultured in an incubator at 37 °C in a humidified atmosphere with 5% CO_2_. Next, 100 μL of HaCaT cell suspension in the complete medium was seeded in each well of a 96-well plate at a density of 1 × 10^5^ cells/mL and incubated for 24 h at 37 °C under 5% CO_2_. Upon 70–80% confluence, the cells were washed once with PBS (pH 7.4). Then, the samples were applied at different concentrations, and the plate was incubated for 24 h. Negative control cells were cells without treatment.

#### 2.9.2. Cytotoxicity Assay

The cytotoxicity assay was examined with MTT dye, in which viable cells can reduce the yellow dye to dark purple formazan crystal by mitochondrial enzymes [26]. The MTT dye was added to cells treated with compounds and incubated for 4 h at 37 °C under a 5% CO_2_ incubator. After incubation, the formazan was then solubilized in 100 μL/well of DMSO, and the absorbance was measured at 570 nm using a microplate reader (Synergy H4, Biotek, Winooski, VT, USA). The absorbance values show a direct proportion with the number of viable cells allowed for quantitative analysis of cytotoxicity.

#### 2.9.3. Intracellular Reactive Oxygen Species (ROS) Generation

Intracellular ROS were identified using an oxidation-sensitive fluorescent probe dye called 2′,7′-dichlorodihydrofluorescein diacetate (H2DCFDA; Ex/Em = 495 nm/529 nm) (Invitrogen Molecular Probes, USA) [27]. The cells were plated in a 96-well plate as described earlier. Of note, 5 μg/mL of 5-fluorouracil (5-FU) was used as an ROS inducer [28,29]. In addition, 20 μM H2DCFDA was added to each well of the 96-well plate, and the plate was incubated at 37 °C for 30 min. The fluorescence was measured at excitation/emission of 485/525 nm with a fluorescence microplate reader (Synergy H4, Biotek, USA) [30].

#### 2.9.4. Scratch Assay

The HaCaT cell line was cultured in 6-well plates (Falcon, Corning Inc., Corning, NY, USA) at a seeding density of 4 × 10^4^ cells/well and allowed to grow up to 85–90% confluence. A scratch was made across the cell monolayer using a 100 μL sterile pipette tip. PBS was used to rinse the scratched cell layer to remove the detached cells. Next, 1 mL of the samples in PBS was added to the well of the scratched cell layer. The plate was incubated, and images were taken with an inverted light microscope (Olympus ΙX-81; Olympus Corporation, Tokyo, Japan) equipped with a camera (EOS 700D, Canon Inc., Tokyo, Japan) at 0, 24, and 48 h after sample loading. Of note, 10 ng/mL TGFβ was used as a positive wound closure control. Each experiment was performed in triplicate, and image acquisition was carried out with analysis using IT software (version 2.3, Olympus Soft Imaging Solutions GmbH, Münster, Germany). Scratch area was measured using Image J software (version 1.53t, National Institutes of Health, Bethesda, MD, USA) [31,32].

### 2.10. Statistical Analysis

PRISM software (GraphPad Prism version 9.5.1, GraphPad Software, San Diego, CA, USA) was used to analyze the data. The results are presented as mean values and standard deviations (SDs). One-way analysis of variance (one-way ANOVA) followed by post hoc Duncan’s tests was performed to assess statistical significance at *p* < 0.05.

## 3. Results

### 3.1. Identification of Crude Saponins of C. oleifera Extracts via LC–QTOF-MS

**Figure 4:**
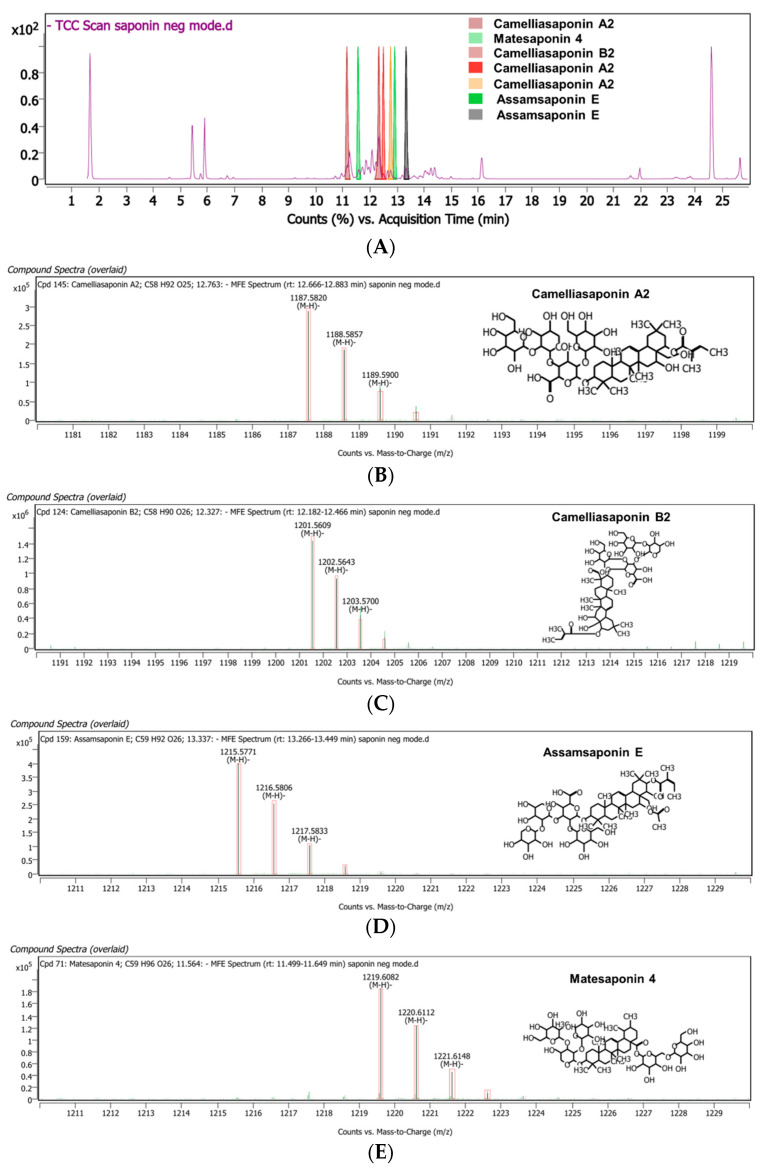
Chromatogram of TS analyzed via LC-QTOF-MS. (**A**) LC-QTOF-MS chromatogram of crude saponins; (**B**) mass spectrum of Camelliasaponin A2 ((M-H)- = 1187.5820); (**C**) mass spectrum of Camelliasaponin B2 ((M-H)- = 1201.5609); (**D**) mass spectrum of Assamsaponin E ((M-H)-= 1215.5771); (**E**) mass spectrum of Matesaponin 4 ((M-H)-= 1219.6082).

Seven saponin compounds were detectable via LC-QTOF-MS. The negative sample mode suggested that seven peaks had matching scores > 85%. The four major compounds found in the sample were Camelliasaponin A2 with three isoforms, Camelliasaponin B2, Assamsaponin E with two isoforms, and Matesaponin 4 (Figure 4) (Table 1).

### 3.2. Antioxidant Activity of TS Extracts Using DPPH and ABTS Assays

The antioxidant activity of TS was determined using DPPH and ABTS assays, which revealed significant free radical scavenging properties. The IC50 values of 66.06 μg/mL were taken from the DPPH assay and 44.75 μg/mL from the ABTS assay. TS at the highest concentration of 125 μg/mL demonstrated notable antioxidant activity, achieving 83.46 ± 0.64% inhibition according to the DPPH assay and 79.41 ± 1.74% from the ABTS assay. However, the antioxidant potency of TS was lower than that of the positive controls, ascorbic acid and Trolox, which exhibited much lower IC50 values (Table 2).

### 3.3. Characterization of Crude Tea Saponin-Incorporated Silica Nanoparticles (TSNPs)

**Figure 5:**
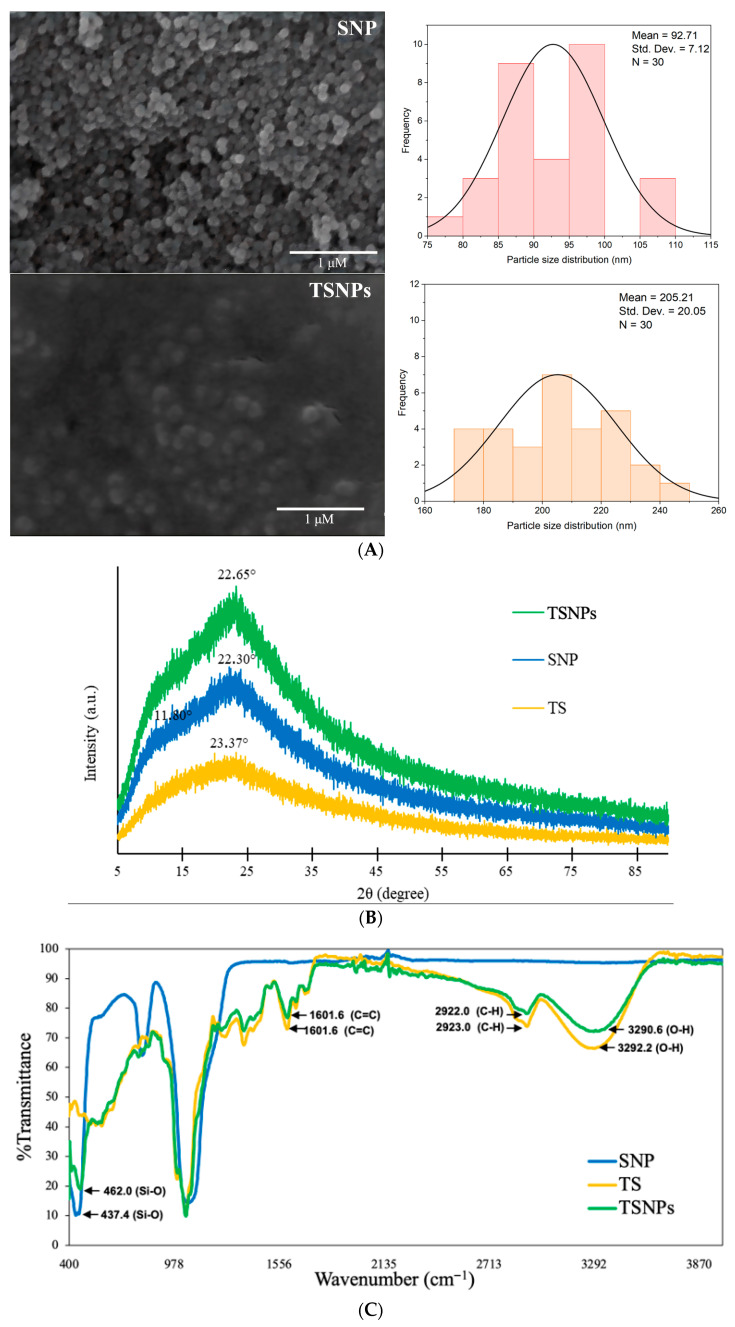
Physicochemical properties of TS, SNPs, and TSNPs. (**A**) Representative images from SEM of SNPs and TSNPs at a magnification of 30,000× with a 1 μm scale bar. The diameter distribution histograms of SNPs and TSNPs were created from measurements from 30 random particle fields (N = 30). (**B**) XRD analysis of TS, SNPs, and TSNPs for crystalline structure determination. (**C**) FTIR spectra of SNPs, TS, and TSNPs. The arrows indicate the main vibrational bands and their assignments: Si-O stretching shifted from 437.4 cm^−1^ (SNPs) to 462.0 cm^−1^ (TSNPs), confirming modifications in the silica framework after TS incorporation; O-H stretching at 3292.2 cm^−1^ (TS) and 3290.6 cm^−1^ (TSNPs), evidencing hydrogen bonding between hydroxyl (-OH) groups of TS and silanol (-SiOH) groups of SNPs; C-H stretching at 2922.0–2923.0 cm^−1^; and C=C stretching at 1601.6 cm^−1^. These characteristic peaks confirm both the integration of TS into the silica matrix and the retention of organic saponin structures in TSNPs.

The aim of this study was to fabricate and characterize TSNPs to understand their structural, morphological, and physicochemical properties for potential optimization in biomedical applications. The silica nanoparticles (SNPs) and TSNPs were investigated by using SEM, Zetasizer, XRD, and FTIR (Figure 5).

The investigation into morphology, size, and zeta potential revealed that both SNPs and TSNPs had a spherical shape; however, the size of the TSNP was larger than that of the SNPs. The average size of the SNPs was 92.71 nm, while TSNPs had an average size of 205.21 nm (Figure 5A) as evidenced by SEM imaging. The surface charge and zeta potential measurements showed that TS, SNPs, and TSNPs had moderately negative surface charges, of which the zeta potential observed in TSNPs was the least negative (−26.30 ± 1.18 mV) while that of TS was −24.90 ± 2.65 mV and that of SNPs was −18.23 ± 0.90 mV. It was also confirmed that SNPs, not TSNPs, were nanoparticles, but both exhibited polydispersity. The diameter measurement from Zetasizer analysis confirmed that SNPs and TSNPs had average diameters of 80.07 ± 26.88 nm and 250.30 ± 29.17 nm, respectively. The polydispersity index (PDI) indicated that both show polydispersity; however, TS had extreme polydispersity (PDI > 0.7) (Table 3).

These findings are significant for the understanding of nanoparticle interactions in biological environments, influencing factors like cellular uptake and therapeutic efficacy. Further research is needed to optimize the potential use of these nanoparticles for biomedical applications.

Hydrogen bonding could indeed play a role in the formation of spherical particles, especially when compounds contain functional groups capable of hydrogen bonding, such as silica and glycoside compounds. Silica is known for its ability to form various structures due to its hydrogen bonding capacity. When glycosidic compounds, which often contain hydroxyl groups capable of forming hydrogen bonds, are introduced into a system containing silica, they can interact via hydrogen bonding forces, potentially leading to the formation of spherical particles or other structures [33,34]. Understanding of these properties is crucial for the prediction of nanoparticle behavior in biological environments [35]. For instance, surface charge, size distribution, and uniformity can influence cellular uptake, biodistribution, and therapeutic efficacy. Further investigations into the correlation of these properties with specific biomedical applications, such as drug delivery or imaging, are essential for optimizing nanoparticle formulations for targeted therapeutic or diagnostic purposes [36].

XRD analysis of SNPs, TS, and TSNPs revealed no differences in the crystallographic properties among them, especially between TS and TSNPs (Figure 5B). TS demonstrated a high degree of crystallinity at 2θ = 23.37° while SNPs appeared at 2θ = 22.30° and at 2θ = 11.80°. In the case of TSNPs, the crystalline structure was observed at 2θ = 22.65°. Our results were consistent with the XRD patterns for all SNP samples, which displayed a broad peak spanning 2θ = 22.3°, indicating the characteristic of amorphous silica [37]. The XRD analysis shows that the incorporation of TS and SNPs to form TSNPs results in a composite material with distinct crystalline characteristics. TSNPs retain the core structure of SNPs, as evidenced by the dominant peak at 22.65°, similar to the peak of SNPs at 22.30° [38]. However, the slight peak shift and increased intensity in TSNPs indicate structural changes due to the interaction between TS and SNPs, leading to a more ordered crystalline phase. TS influences the crystal structure without drastically altering it, resulting in a composite with improved crystallinity compared to TS alone [39]. TSNPs present as a new material that exhibits properties of both TS and SNPs while forming a distinct crystalline phase [40].

The FTIR spectra of TS, SNPs, and TSNPs confirmed the successful incorporation of tea saponins (TS) into silica nanoparticles (SNPs) through distinct chemical interactions. The Si-O stretching band at 437.4 cm^−1^ (SNPs) shifted to 462.0 cm^−1^ (TSNPs), indicating structural modifications in the silica network due to the presence of TS. The broad O-H stretching (3292.1 cm^−1^ in TS and 3290.6 cm^−1^ in TSNPs) suggests hydrogen bonding interactions between the hydroxyl (-OH) groups of TS and silanol (-SiOH) groups of SNPs, further supporting successful TS incorporation into TSNPs. Additionally, C-H stretching at 2922.8 cm^−1^ (TSNPs) and 2923.0 cm^−1^ (TS) confirms the retention of organic saponin chains. The C=C stretching band at 1602.7 cm^−1^ (TSNPs) and 1601.6 cm^−1^ (TS) indicates the presence of aromatic rings from TS, showing that its structural properties remain intact after nanoparticle integration (Figure 5C). These spectral shifts and peak retention indicate that TS was successfully embedded within SNPs, forming TSNPs with distinct surface chemistry and improved molecular interactions.

Finally, we successfully fabricated TSNPs. SEM and Zetasizer analyses revealed that TSNPs had a spherical morphology significantly larger than SNPs, averaging 205.21 nm versus 92.71 nm. Zeta potential measurements indicated moderately negative surface charges, with TSNPs showing the least negative value of −26.30 mV. XRD patterns confirmed the incorporation of crude tea saponin into the silica framework, resulting in a more ordered crystalline phase. FTIR analysis further demonstrated hybrid surface chemistry, with shifts in Si-O stretching indicating enhanced TS integration. These findings suggest that TSNPs possess properties suitable for biomedical applications, such as targeted drug delivery or imaging.

### 3.4. Quantitative Analysis of Saponin in TSNPs

To quantify the saponin equivalent concentration in TS and TSNPs, we used HPLC, though no standard saponin was available for direct calibration in this study. This study quantified the saponin concentration in TS and TSNPs using HPLC and LC-QTOFMS, identifying seven saponin compounds (Table 1) in TS and confirming the presence of saponin at the principal peak of 26.4 min in the HPLC chromatogram. A calibration curve was established based on a 45% purity of saponin in TS. This study developed a saponin equivalency model linking peak area and saponin concentrations. This model allowed us to compare biological activities between TS and TSNPs reliably. The 3D plot underscored the correlation between saponin concentration, peak area, and concentration, supporting consistent dosing in in vitro studies.

**Figure 6:**
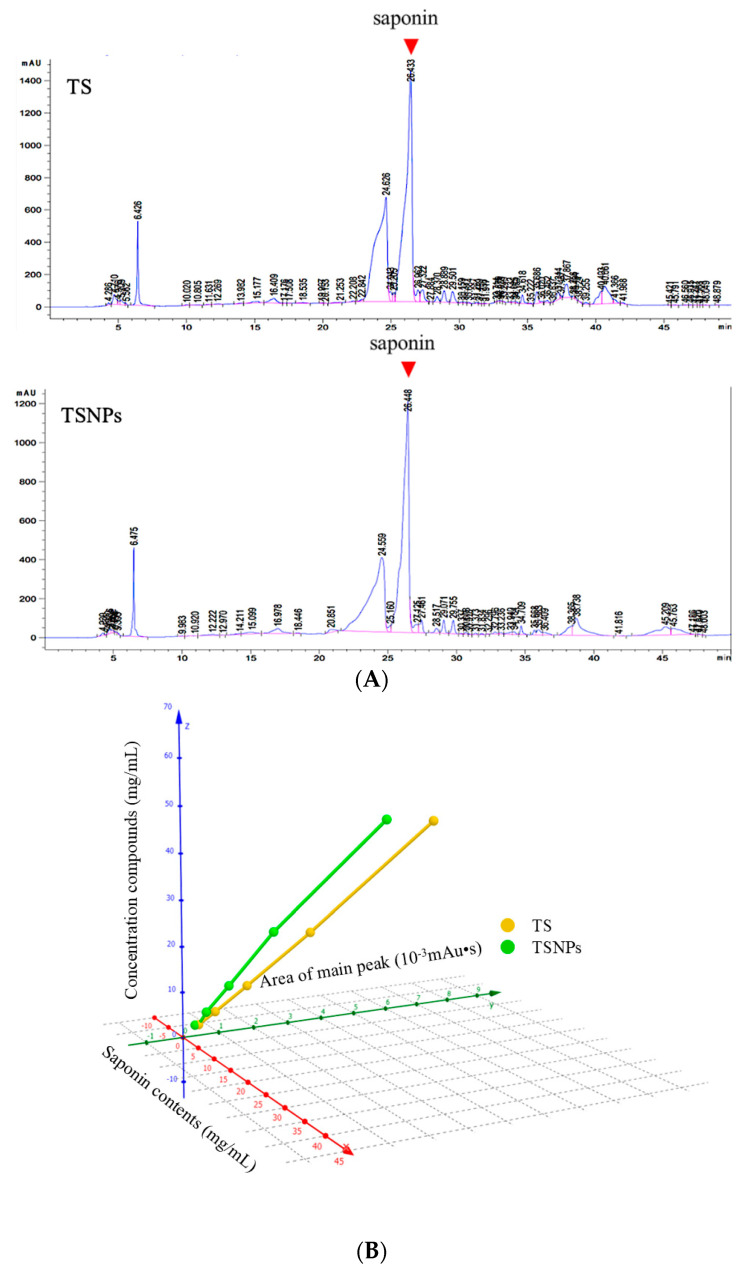
Determination of saponin concentration. HPLC chromatograms of TS and TSNPs (**A**) and a 3D plot of saponin concentration (mg/mL), area of the main peak (10^−3^ mAu·s), and the concentration of either TS or TSNPs (**B**).

The HPLC chromatogram revealed three prominent peaks in both TS and TSNPs at retention times of 6.4, 24.6, and 26.4 min, with the latter identified as the principal peak (Figure 6A). This finding is in alignment with previous studies wherein saponin compounds displayed sharp peaks within the retention time range of 26.4 to 26.8 min, confirming the presence of saponin in our samples [2,22]. To estimate the saponin equivalent concentration in TS and TSNPs, we used 50 mg/mL TS for the HPLC analysis, and the peak area at 26.4 min in the chromatogram was approximated for a saponin concentration using 45% purity. This value suggests that a 50 mg/mL (Z_TS_) solution of TS corresponds to a saponin concentration of 22.5 mg/mL (X^S^ = 0.45 × Z_TS_). A concentration curve of saponin in TS relative to peak area was developed for saponin concentrations in TS to approximate the saponin concentration in TSNPs at different prepared concentrations (Supplementary Table S1). The data were plotted in a 3-parameter plot (Figure 6B), and the equations between the saponin concentration (X_S_) and the TS concentration (Z_TS_) or the TSNP concentration (Z_TSNPs_) were extrapolated for preparation of the TS (Equation (1)) and TSNPs (Equation (2)) at equivalent saponin concentration.

Z_TS_ = 2.2223X_S_ − 0.0017 (R^2^ = 1) (1)

Z_TSNPs_ = 2.6087X_S_ + 0.0569 (R^2^ = 0.9985) (2)

This saponin equivalency calculation is essential for in vitro studies (Supplementary Table S2), as it ensures consistent dosing when comparing the effects of TS and TSNPs. This study provides a reliable foundation for comparing biological activity between TS and TSNPs by validating the equivalent saponin concentration in both formulations.

### 3.5. In Vitro Release of Saponins from TS and TSNPs

The in vitro release of saponins from TS and TSNPs was evaluated based on cumulative percentage release over 48 h. It was found that saponins were significantly released from TS within 30 h (*p* < 0.05) of investigation. After 30 h of submersion, the cumulative concentrations of saponins from both TS and TSNPs were similar.

**Figure 7:**
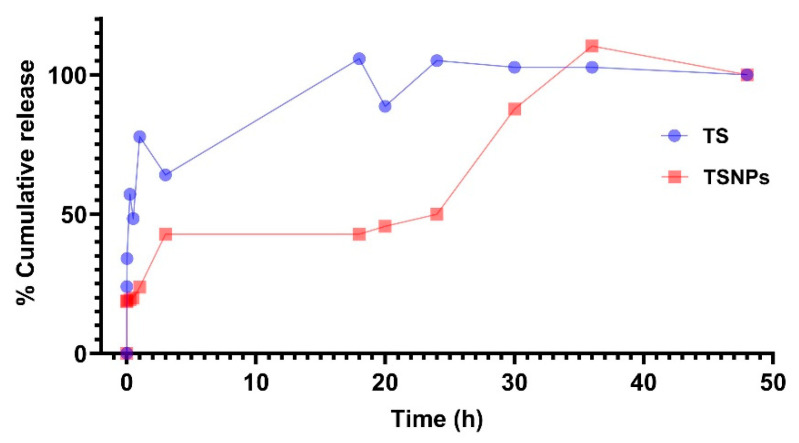
In vitro cumulative release of saponins from TS and TSNPs. Tea saponin (TS) and TS-incorporated nanoparticles (TSNPs) were submerged in 80% ethanol for 48 h.

The values of t_50_ and t_90_ of saponins in TS values were determined as 30 min and 20 h, whereas those of saponin TSNPs were 24 h and 30 h, respectively. From this result, it was confirmed that TSNPs had sustained-release characteristics of the nanoparticle formulation when compared to TS. In other words, these findings confirmed that silica nanoparticle encapsulation modified the release kinetics of tea saponin from a burst-release to a sustained-release profile, which is advantageous for prolonging therapeutic exposure, minimizing dosing frequency, and reducing cytotoxicity (Figure 7).

### 3.6. Enhanced Biocompatibility and Reduced Cytotoxicity of TSNPs Compared to Crude Tea Saponin

Through cytotoxic study of HaCaT cells, we found that the incorporation of TS with SNPs notably reduced the cytotoxicity of TS. SNPs exhibited the least cytotoxicity with an IC_50_ of 909.9 μg/mL, while TS showed the greatest cytotoxicity with an IC_50_ of 33.5 μg/mL (15.1 μg/mL saponin equivalency from Equation (1)). In contrast, TSNPs had an IC_50_ of 60.3 μg/mL (23.1 μg/mL saponin equivalency from Equation (2)).

**Figure 8:**
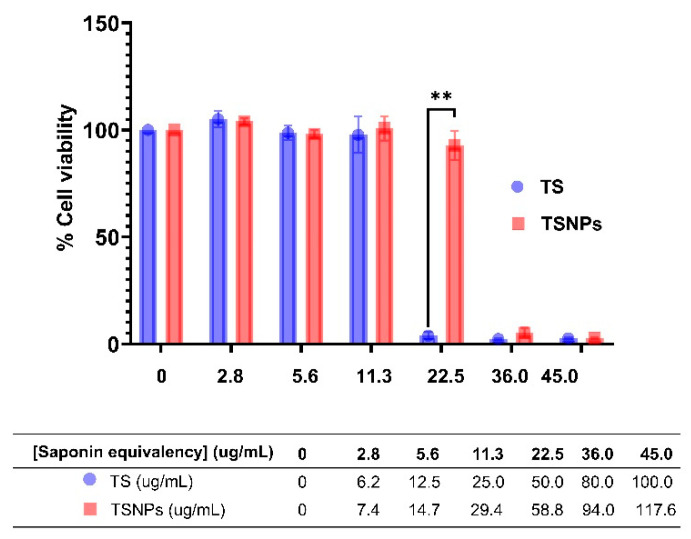
The percentage of cell viability of HaCaT cells cultured with TS and TSNPs for 24 h at different concentrations is based on the saponin equivalent concentration. ** denotes a statistically significant difference when *p* < 0.01.

TSNPs maintained greater cell viability across all tested saponin-equivalent concentrations, with a statistically significant difference observed at 22.5 µg/mL (*p* < 0.001). However, a statistically significant reading was found for both TS and TSNPs at saponin concentration equivalency of 36.0 and 45.0 µg/mL and above (Figure 8).

Our findings provide evidence that TSNPs improved the safety of saponins. Even though SNPs were largely non-toxic, they lacked the functional activity associated with TS. Overall, the incorporation of TS into SNPs not only reduced toxicity but also retained functional efficacy, leading to the conclusion that TSNPs could be a promising candidate for biomedical applications in the treatment of dental diseases.

### 3.7. Enhanced Antioxidant Potential of Saponin-Based Nanoparticles (TSNPs) in Mitigating Oxidative Stress Induced by 5-Fluorouracil in HaCaT Cells

**Figure 9:**
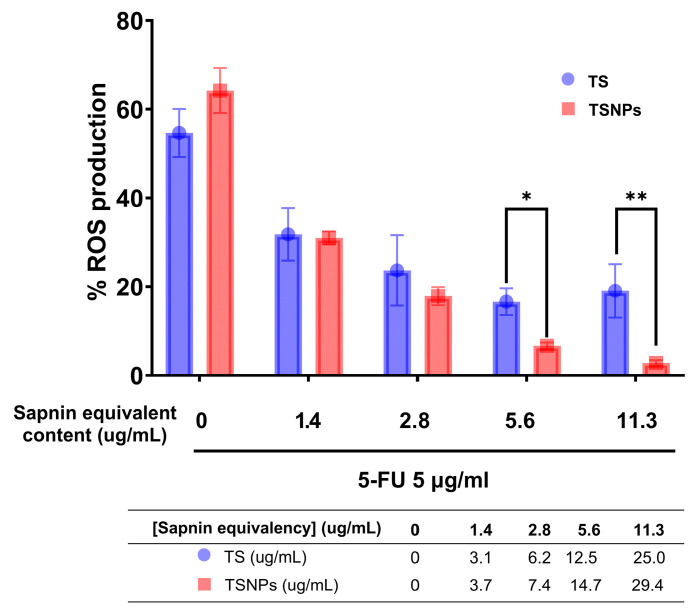
Percentage of ROS production in HaCaT cells cultured with TS and TSNPS for 24 h at various saponin equivalent concentrations. 5-fluorouracil (5-FU) was used as an inducer for the generation of ROS. The percentage production of ROS was compared with normal cells without 5-FU treatment. ** and * denote that the results showed a statistically significant difference when *p* < 0.01 and *p* < 0.05, respectively.

Both TS and TSNPs exhibited antioxidant properties in 5-fluorouracil (5-FU)-induced HaCaT cells, indicating the anti-oxidative stress properties of both compounds. TS and TSNPs at different equivalent saponin concentrations were tested, and it was found that a concentration as low as 1.4 µg/mL of saponin in both TS and TSNPs significantly reduced ROS levels in HaCaT cells. ROS levels remained unchanged when increasing saponin concentrations from 2.8 to 11.3 µg/mL in the TS, while the ROS levels were significantly reduced when saponin concentrations were increased from 1.4 to 11.3 µg/mL in the TS in a dose-dependent manner (Figure 9).

### 3.8. Enhanced Cell Migration with Crude Tea Saponin-Incorporated Nanoparticles (TSNPs)

**Figure 10:**
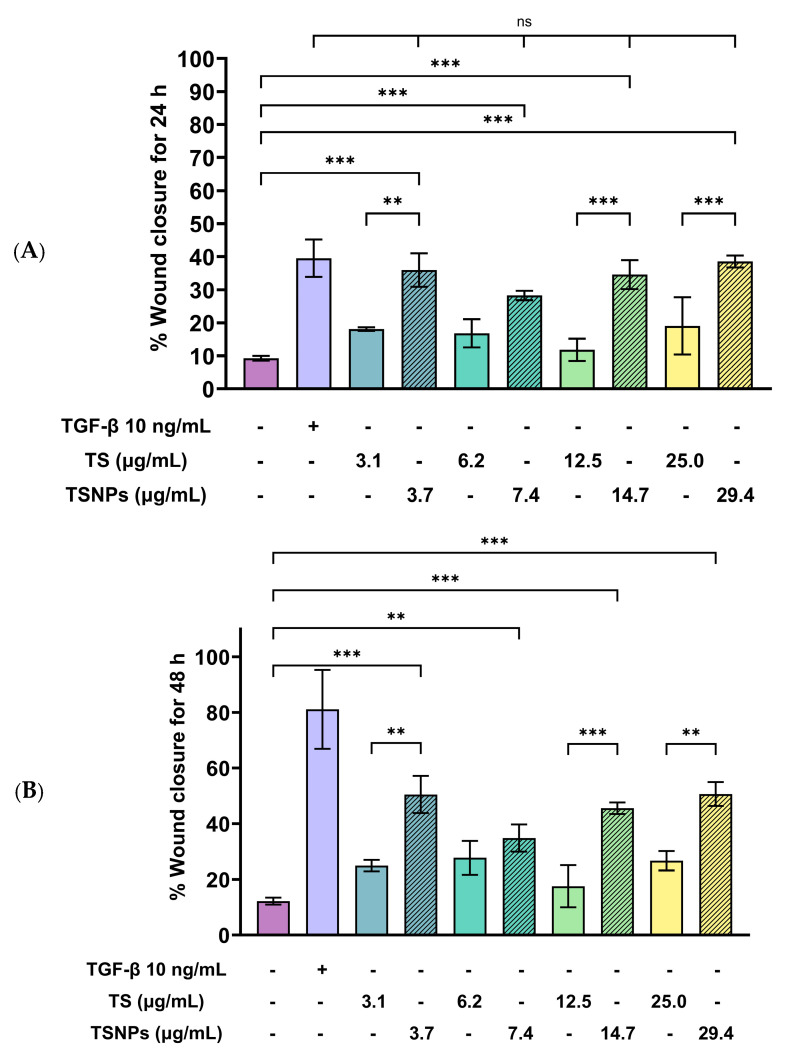
This study examines the effects of TS and TSNPs on wound healing in HaCat cells by assessing wound closure in a scratch assay at 24 and 48 h. HaCat cells were untreated (negative control) or treated with TS at concentrations of 3.1–25.0 μg/mL or TSNPs at 3.7–29.4 μg/mL. Bar graphs of similar filled colors represent equivalent saponin concentrations, solid and striped bars correspond to varying TS and TSNP concentrations, respectively. TGF-β (10 ng/mL) was used as a positive control. (**A**,**B**) Data show that TSNPs were more effective in promoting wound closure than TS and the control (*p* < 0.005) at 24 and 48 h, especially at concentrations of 3.7, 14.7, and 29.4 μg/mL. The highest wound closure (~80%) was observed in the TGF-β group after 48 h. Data are presented as the mean ± SD, with significance tested via one-way ANOVA followed by Tukey’s post hoc test (** *p* < 0.01; *** *p* < 0.001; ns = not significant). Bar colors represent equivalent saponin concentrations, while striped bars indicate TSNPs samples.

To evaluate the effects of tea saponins (TS) and their nanoparticle formulation (TSNPs) on wound healing, a scratch wound healing assay was performed using various concentrations of each agent. Wound closure was assessed at 0, 24, and 48 h, and cell migration was quantified. Microscopic images revealed differences in the dynamics of wound closure among the experimental groups (Table 4), which were confirmed through semi-quantitative analysis at 24 and 48 h (Figure 10)

The untreated control group exhibited minimal wound closure over the entire 48 h period. In contrast, the positive control group, treated with TGF-β (10 ng/mL), showed significant wound healing, with 80% closure at 48 h (*p* < 0.001). Treatment with TS resulted in a plateau effect, whereby only modest improvement in wound closure was observed. A dose-dependent pattern was evident at higher TS concentrations, which actually reduced cell migration. At a concentration of 25.0 μg/mL, the wound closure at 48 h was below 30%.

Remarkably, TSNPs demonstrated greater wound healing efficacy than TS and similar efficacy to TGF-β at 24 h. The nanoparticle formulation consistently outperformed TS at equivalent saponin concentrations. For example, TSNPs at 29.4 μg/mL achieved nearly 60% wound closure at 48 h, significantly higher than the 25.0 μg/mL TS treatment, which showed less than 30% closure (*p* < 0.01). Additionally, TSNPs accelerated wound healing at earlier time points. At 24 h, a substantial amount of wound closure was already observed in the TSNP-treated group, particularly at concentrations of 14.7 and 29.4 μg/mL (*p*-values ranging from <0.01 to <0.001). However, the positive effects of TSNPs were significantly lower than those of TGF-β treatment at 48 h (Figure 10).

In conclusion, TSNPs enhanced wound healing in a dose- and time-dependent manner. TS appears to have limitations due to poor solubility and reduced cellular uptake. Encapsulating TS within nanoparticles addresses these challenges. These results support the hypothesis that delivering plant-derived compounds through nanoparticles improves their biocompatibility and bioactivity, confirming their potential for clinical use in wound healing.

## 4. Discussion

The integration of TS into SNPs significantly advances the delivery of bioactive compounds. The embedding of saponin micelles into silica nanoparticles enhanced stability and bioactivity [41,42]. Similarly, the use of silica nanoparticles as carriers for bioactive compounds such as flavonoids, phenolic acids, and saponins demonstrated improved cellular uptake and therapeutic efficacy [14,43]. TS exhibited notable antioxidant activity (IC50: 66.06 µg/mL in DPPH; 44.75 µg/mL in ABTS). The difference in IC_50_ values observed between the DPPH and ABTS assays is not large and can be explained by the chemical characteristics of the radicals. It is well known that the DPPH radical is lipophilic and mainly reacts with hydrophobic antioxidants, while the cationic ABTS•^+^ radical is hydrophilic. TS is a natural surfactant that is soluble in both aqueous and organic media, enabling interaction with a wider range of hydrophilic and lipophilic antioxidant compounds. Therefore, the lower IC_50_ value observed in the ABTS assay suggests that TS contains hydrophilic antioxidant constituents that are more efficiently detected by ABTS than by DPPH, comparable to *Vernonia amygdalina* (50–70 µg/mL) and *Camellia sinensis* (40–60 µg/mL). *C. sinensis* showed slightly stronger ABTS activity due to higher polyphenolic content, while *Vernonia amygdalina* had a closer capacity to TS in DPPH assays. These variations reflect differences in saponin and polyphenol ratios and assay conditions [18,20].

Nanoparticles fabricated with tea saponins showed particle sizes in the range of 205.21 ± 20.05 nm (SEM) and 250.30 ± 29.17 nm (Zetasizer, Malvern Instruments Ltd., Worcestershire, UK), consistent with reported sizes for silica-based nanoparticles. These dimensions are suitable for biomedical applications, as nanoparticles with diameters ranging between 200 and 300 nm and a zeta potential of approximately −25 mV are ideal for cellular uptake and therapeutic delivery [44]. The TSNPs fabricated in this study displayed a mean diameter of 205.21 ± 20.05 nm and a zeta potential of −26.30 ± 1.18 mV, which are in the optimal ranges for biomedical use. X-ray diffraction analysis indicates subtle structural changes in the nanoparticles, with a characteristic amorphous silica peak near 2θ = 22° and a slight shift to 22.65° upon saponin incorporation, suggesting enhanced structural ordering [13]. The incorporation of tea saponins (TS) into silica nanoparticles (SNPs) influences the crystallinity of the composite without significantly altering its core structure. This effect is due to the rearrangement of hydrogen bonding, molecular templating, and the silica network. Hydroxyl (-OH) and glycosidic groups in TS interact with Si-OH groups of silica, modulating the siloxane (Si-O-Si) framework and subtly modifying X-ray diffraction (XRD) patterns. Similar templating effects have been reported in polyphenols, flavonoids, and hybrid nanocomposites, wherein organic molecules guide crystallite alignment and enhance structural ordering. The XRD analysis in this study revealed a slight peak shift and increased crystallinity in TSNPs compared to TS alone, suggesting structural reorganization within the silica framework. Other studies support the concept that organic–inorganic hybrid materials exhibit improved crystallinity due to molecular interactions [38,40]. FTIR analysis confirmed distinct chemical interactions between TS and SNPs, primarily through shifts in Si-O stretching vibrations. This suggests hydrogen bonding between hydroxyl (-OH) groups of TS and silica (-SiOH) functional groups of SNPs. It has been reported that the incorporation of phytochemicals into silica nanoparticles induces similar spectral shifts due to strong intermolecular interactions [45]. In the case of TSNPs, the Si-O stretching vibration shifted from 437.4 cm^−1^ (SNPs) to 462.0 cm^−1^ (TSNPs), indicating structural modifications within the silica network due to the successful incorporation of TS. Details pertinent to this shift are in alignment with a previous study on mesoporous silica nanoparticles (MSNs) loaded with bioactive phytoconstituents, whereby hydrogen bonding and surface functionalization led to comparable vibrational shifts [46]. Additionally, other phytochemical-functionalized silica nanostructures, such as those incorporating flavonoids and triterpenoids, have exhibited similar Si-O vibrational changes, confirming hydrogen bonding and potential covalent interactions within the silica framework [47]. These interactions enhance nanoparticle stability, improve biological functionality, and contribute to the efficacy of TSNPs for biomedical applications, particularly in the case of controlled drug release and antioxidant therapies [48].

Saponin purity in tea saponin nanoparticles is quantified at 45% using HPLC, with calibration models based on crude extract standards addressing the absence of direct standards [23,49]. Saponins extracted from other plants, such as *Quillaja saponaria* and *Panax ginseng*, often achieve purity levels ranging from 40% to 60% using solvent-based extraction methods, including ethanol or methanol precipitation [2]. Comparatively, the 45% purity observed in tea saponins was within this range, though slight variations may arise from differences in solvent systems and extraction techniques [49]. In contrast, the use of aqueous ethanol in ginseng yields higher purities but requires longer processing times [49]. Understanding these variations helps optimize the extraction process for specific applications.

TSNPs exhibited a significantly different release profile of saponins compared to TS, which is consistent with a sustained-release mechanism. TS demonstrated a burst release. In contrast, TSNPs showed a slower release under the control of saponin concentration of 11.30 mg/mL in both TS and TSNPs. We propose that TSNPs exhibited releasing behaviors as a mixture between absorption and encapsulation due to the burst release at the very beginning and the presentation of lag, log, and stationary phase of release at the late time point. In addition, this explanation could be supported by FTIR results and the SEM, as well as Zetasizer, exhibiting an increase in diameter of the TSNPs when compared with SNPs. These findings highlight the ability of the silica nanoparticle matrix to modulate drug diffusion, prevent burst release, and maintain prolonged drug availability, which is critical for therapeutic efficiency and reduced toxicity [50]. However, the present investigation primarily focused on short-term physicochemical characterization and in vitro release kinetics within 48 h. While these findings provide important insights into the immediate stability and functionality of TSNPs, the question of long-term storage stability remains open. For instance, evaluating whether particle size, zeta potential, or morphological features undergo significant alterations during extended storage periods would be critical to ensure their translational applicability. Future studies should therefore address the systematic monitoring of these parameters under different storage conditions to establish the robustness, shelf life, and clinical potential of TSNPs.

Cytotoxicity assays revealed that TSNPs reduced toxicity compared to TS, with IC_50_ values of 60.3 µg/mL and 33.5 µg/mL, respectively. The assays were conducted using HaCaT cells, a widely used human keratinocyte cell model that is particularly relevant for the evaluation of skin-related biocompatibility and toxicity [30]. The findings were in alignment with previous studies, indicating that nanoparticle-based delivery systems enhance biocompatibility by modulating release kinetics. The silica nanoparticle matrix in TSNPs may control saponin release and reduce the peak of bioactive compound concentrations that may alter cellular stress conditions. Additionally, TS encapsulation enhances stability and minimizes direct saponin-membrane interactions, further mitigating cytotoxicity [51,52]. The MTT assay results in this study support these findings, as TS exhibited significant cytotoxicity when compared to TSNPs at the saponin concentration of 22.5 µg/mL. This effect is attributed to controlled release and reduced direct exposure to free saponin-containing TS. This finding highlights the protective role of TSNPs in mitigating TS-induced cytotoxicity.

The observed reduction in intracellular ROS levels with increasing concentrations of TS and TSNPs can be attributed to their antioxidant activity. Tea saponins contain functional groups capable of scavenging free radicals, thereby mitigating oxidative stress. For example, green tea-derived saponins showed ROS scavenging and activation of antioxidant response in HEK293 cells [53]. Moreover, the incorporation of TS into silica nanoparticles enhances their stability, loading capacity, and cellular uptake, thereby improving delivery and resulting in more efficient ROS neutralization at higher concentrations (functionalized silica nanoparticles have been reported to enhance antioxidant performance via improved delivery) [54]. These findings support the role of TS and TSNPs as potential antioxidative agents for protecting cells against oxidative damage.

TSNPs outperformed TS in cell migration, with enhanced wound closure rates. This was consistent with previous research on saponin-based nanoparticles, which reported improved cell migration leading to wound healing due to the controlled release of active compounds [55,56]. Saponins are known to promote wound healing by modulating key signaling pathways, such as TGF-β (transforming growth factor-β), VEGF (vascular endothelial growth factor), and NF-κB (nuclear factor kappa-light-chain-enhancer of activated B cells). These pathways are crucial for cellular proliferation, angiogenesis, and the inflammatory response, which collectively accelerate tissue regeneration. For example, TGF-β signaling facilitates the migration and differentiation of fibroblasts essential for the formation of new extracellular matrix (ECM). Likewise, VEGF promotes the formation of new blood vessels and ensures adequate oxygen and nutrient supply to the wound site [55].

Other phytochemicals, such as flavonoids (e.g., quercetin) and triterpenoids (e.g., asiaticoside from *Centella asiatica*), exhibited similar wound-healing mechanisms. Quercetin enhances keratinocyte migration and proliferation via the PI3K/Akt pathway, while asiaticoside upregulates TGF-β signaling to promote fibroblast activity and collagen synthesis. These molecular mechanisms have been thought to be parallel to those observed in saponins, highlighting their role in accelerating tissue repair. The incorporation of saponins into nanoparticles, as in TSNPs, potentially enhanced these effects through sustained release, the maintenance of therapeutic concentrations at the wound site, and the minimization of cytotoxicity. This controlled release mechanism ensures prolonged activation of the TGF-β and VEGF pathways to enhance wound healing [55,56,57,58].

Apart from wound healing benefits, TSNPs may also represent a promising platform for cancer therapy. Tea saponins have demonstrated anticancer activities in several cancer types, including breast, colorectal, hepatocellular, lung, and skin cancers, through mechanisms such as apoptosis induction, oxidative stress modulation, and angiogenesis inhibition. Incorporation of tea saponins into silica nanoparticles may enhance these therapeutic effects by improving their bioavailability, prolonging release, and minimizing systemic toxicity [59,60,61,62,63]. Such improvements could be particularly valuable in therapeutic contexts such as adjunct chemotherapy, topical or locoregional therapy (e.g., skin and oral cancers), and chemoprevention in high-risk populations (e.g., colorectal cancer predisposition). Therefore, TSNPs represent a promising nanoplatform with potential applications across multiple cancer types and therapeutic settings.

## 5. Conclusions

This study has successfully demonstrated the development of TSNPs exhibiting significantly enhanced biological properties compared to TS. In particular, TSNPs showed significantly reduced cytotoxicity in comparison with TS at the equivalent saponin concentration by significantly reducing ROS production due to a controlled release behavior. In addition, the statistically significant promotion of wound healing was observed in TSNP-treated cells in all tested concentrations at equivalent saponin concentrations. When compared with TGFβ treatment, TSNPs had similar efficiency in promoting wound closure at an early time point. Moreover, the physical properties of TSNPs were not significantly different from those of the SNPs, apart from the size and the presence of saponin on their surface. This fabrication of TSNPs could improve the biological effects of using SNPs and saponin in medical applications. Notably, the enhanced effectiveness and bioavailability of these TSNPs offer promising avenues for their use in chemotherapeutic treatments and wound healing applications, showcasing a significant step forward in developing safer and more efficient bioactive compounds.

## Figures and Tables

**Table 1:** Seven saponins identified in crude tea saponin extract according to LC-QTOF-MS.

No.	Compounds Name	Structure	RT	Matching Score (%)	*m*/*z*	(M-H)-	Mass	Mass Diff (Tgt/ppm)
1	Camelliasaponin A2: Cpd 145	C_58_H_92_O_25_	12.76	92.83	1187.58	1187.58	1188.59	−2.46
2	Camelliasaponin A2: Cpd 54	C_58_H_92_O_25_	11.15	91.13	1233.59	1233.59	1188.59	−3.15
3	Camelliasaponin A2: Cpd 136	C_58_H_92_O_25_	12.49	85.31	1187.58	1187.58	1188.59	−2.61
4	Camelliasaponin B2: Cpd 124	C_58_H_90_O_26_	12.33	89.97	1201.56	1201.56	1202.57	−2.60
5	Assamsaponin E: Cpd 159	C_59_H_92_O_26_	13.34	93.07	1215.58	1215.58	1216.58	−2.71
6	Assamsaponin E: Cpd 151	C_59_H_92_O_26_	12.92	85.44	1215.58	1215.58	1216.58	−2.85
7	Matesaponin 4: Cpd 71	C_59_H_96_O_26_	11.56	90.17	1219.61	1219.6082	1220.61	−2.96

**Table 2:** Antioxidant properties of TS tested using the DPPH and ABTS assays.

TS Concentration (μg/mL)	DPPH			ABTS		
	O.D. 520	% Inhibition	IC_50_	O.D. 734	% Inhibition	IC_50_
15.625	0.81	20.53 ± 1.26	66.06	0.61	13.61 ± 1.62	44.75
31.25	0.67	34.40 ± 0.88		0.39	43.86 ± 0.45	
62.5	0.50	51.28 ± 1.15		0.24	66.24 ± 1.20	
125	0.17	83.46 ± 0.64		0.14	79.41 ± 1.74	

**Table 3:** Zeta potential analyses of TS, SNPs, and TSNPs.

Sample	Zeta Potential (mV)	Average Diameter Calculated via Laser Particle Size Measurement (nm)	PDI	Average Diameter Calculated from SEM (nm, N = 30)
TS	−24.90 ± 2.65	450.00 ± 229.50	0.84	230.26 ± 108.97
		7.55 ± 1.68		
		84.47 ± 28.93		
SNPs	−18.23 ± 0.90	80.07 ± 26.88	0.15	92.71 ± 7.12
TSNPs	−26.30 ± 1.18	250.30 ± 29.17	0.38	205.21 ± 20.05

**Table 4:** The different doses of tea saponins (TS) and tea saponin-incorporated nanosilica particles (TSNPs) were allowed to migrate at the time points 0, 24, and 48 h, and the images were captured in a wound healing assay. For TS, the concentrations ranged from 3.1 to 25.0 μg/mL, and for TSNPs, the concentration range of 3.7 to 29.4 μg/mL was used. Wound closure was assessed at each time point, and the TS groups were compared to control (Clt), the TGF-β 10 ng/mL positive control, and equivalent saponin concentration (1.4, 2.8, 5.6, and 11.3 μg/mL). Cells in the absence of treatment served as negative controls. Images of wound closure by HaCaT cells were visualized by using inverted microscopy at 10× magnification. The scale bar is 100 μm. The scale bar is 100 μm.

Saponin Concentration (μg/mL)	Treatment	0 h	24 h	48 h
-	Clt	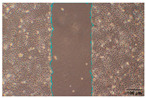	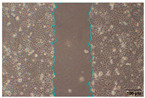	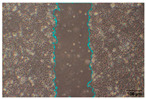
-	TGF-β 10 ng/mL	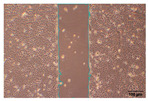	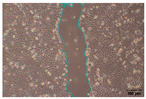	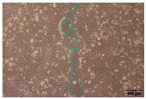
1.4 μg/mL	TS 3.1 μg/mL	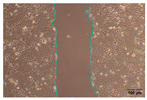	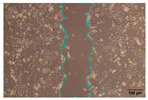	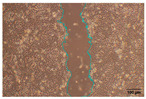
	TSNPs 3.7 μg/mL	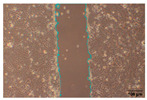	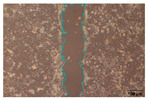	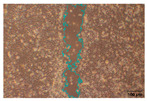
2.8 μg/mL	TS 6.2 μg/mL	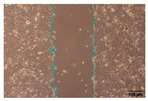	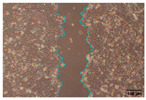	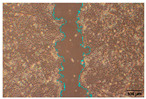
	TSNPs 7.4 μg/mL	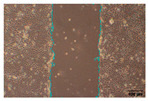	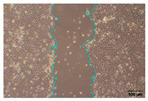	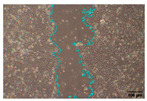
5.6 μg/mL	TS 12.5 μg/mL	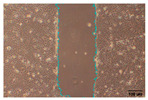	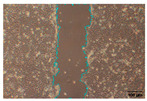	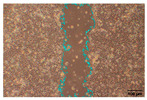
	TSNPs 14.7 μg/mL	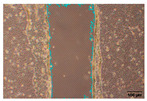	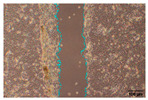	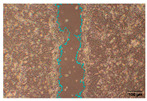
11.3 μg/mL	TS 25.0 μg/mL	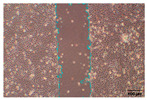	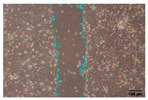	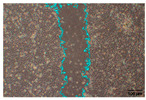
	TSNPs 29.4 μg/mL	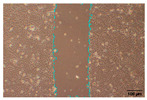	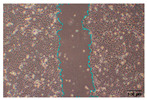	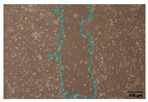
		Scale bar 100 μm		

## Data Availability

The original contributions presented in the study are included in the article, further inquiries can be directed to the corresponding author.

## Supplementary Materials

The following supporting information can be downloaded at: https://www.mdpi.com/article/10.3390/jfb16100390/s1, Table S1: Quantification of saponin content in TS and TSNPs based on HPLC peak area; Table S2: Saponin equivalency in TSNPs relative to TS based on calculated values.

## References

[1] Zhang X., Li C., Hu W., Abdel-Samie M.A., Cui H., Lin L. (2024). An overview of tea saponin as a surfactant in food applications. Crit. Rev. Food Sci. Nutr..

[2] Ahmed H.O.A., Wang C. (2015). Determination of tea saponin in Camellia seed oil with UV and HPLC analysis. World J. Eng. Technol..

[3] Qin P., Shen J., Wei J., Chen Y. (2024). A critical review of the bioactive ingredients and biological functions of *Camellia oleifera* oil. Curr. Res. Food Sci..

[4] Peng X., He X., Tang J., Xiang J., Deng J., Kan H., Zhang Y., Zhang G. (2022). Evaluation of the in vitro antioxidant and antitumor activity of extracts from *Camellia fascicularis* leaves. Front. Chem..

[5] Zhang J., Ying Y., Li X., Yao X. (2020). Changes in tannin and saponin components during co-composting of *Camellia oleifera* Abel shell and seed cake. PLoS ONE.

[6] Li Z., Liu A., Du Q., Zhu W. (2022). Bioactive substances and therapeutic potential of camellia oil: An overview. Food Biosci..

[7] Saridou M., Nikolaidis A.K., Koulaouzidou E.A., Achilias D.S. (2023). Synthesis and characterization of dental nanocomposite resins reinforced with dual organomodified silica/clay nanofiller systems. J. Funct. Biomater..

[8] Jiangkongkho P., Arksornnukit M., Takahashi H. (2018). The synthesis, modification, and application of nanosilica in polymethyl methacrylate denture base. Dent. Mater. J..

[9] Patel A., Patel N., Ali A., Alim H. (2023). Nanomaterials synthesis using saponins and their applications. Secondary Metabolites Based Green Synthesis of Nanomaterials and Their Applications.

[10] Chowdhury M.A. (2016). The controlled release of drugs and bioactive compounds from mesoporous silica nanoparticles. Curr. Drug Deliv..

[11] Karumuri S., Mandava J., Pamidimukkala S., Uppalapati L.V., Konagala R.K., Dasari L. (2020). Efficacy of hydroxyapatite and silica nanoparticles on erosive lesions remineralization. J. Conserv. Dent..

[12] Güçlü Z.A., Patat Ş., Coleman N.J. (2024). The impact of nano- and micro-silica on the setting time and microhardness of conventional glass-ionomer cements. Dent. J..

[13] Simonescu C.-M., Dumitru F., Zărnescu B., Culiță D.C., Răzvan A., Oprea O., Trușcă R., Vasile E. (2024). Competitive adsorption of aqueous Cd(II) and Pb(II) solutions onto silicas synthesized with saponin as template agent. J. Compos. Sci..

[14] Wang D., Sha L., Xu C., Huang Y., Tang C., Xu T., Li X., Di D., Liu J., Yang L. (2022). Natural saponin and cholesterol assembled nanostructures as the promising delivery method for saponin. Colloids Surf. B Biointerfaces.

[15] Casarrubios L., Gómez-Cerezo N., Feito M.J., Vallet-Regí M., Arcos D., Portolés M.T. (2018). Incorporation and effects of mesoporous SiO_2_-CaO nanospheres loaded with ipriflavone on osteoblast/osteoclast cocultures. Eur. J. Pharm. Biopharm..

[16] Liu D., Lei S., Hu Y., Li Z., Xi H., Lin X. (2024). Environmentally friendly tea saponin foam detergents costabilized by GO-OH/SiO_2_ nanoparticles for removing radioactive surface contaminants. Colloids Surf. A Physicochem. Eng. Asp..

[17] Arjin C., Hongsibsong S., Pringproa K., Seel-audom M., Ruksiriwanich W., Sutan K., Sommano S.R., Sringarm K. (2021). Effect of Ethanolic *Caesalpinia sappan* Fraction on In Vitro Antiviral Activity against Porcine Reproductive and Respiratory Syndrome Virus. Vet. Sci..

[18] Brand-Williams W., Cuvelier M.E., Berset C. (1995). Use of a Free Radical Method to Evaluate Antioxidant Activity. LWT-Food Sci. Technol..

[19] Hussen E.M., Endalew S.A. (2023). In Vitro Antioxidant and Free-Radical Scavenging Activities of Polar Leaf Extracts of *Vernonia amygdalina*. BMC Complement. Med. Ther..

[20] Re R., Pellegrini N., Proteggente A., Pannala A., Yang M., Rice-Evans C. (1999). Antioxidant Activity Applying an Improved ABTS Radical Cation Decolorization Assay. Free Radic. Biol. Med..

[21] Elizondo-Villarreal N., Gandara-Martínez E., García-Méndez M., Gracia-Pinilla M., Guzmán-Hernández A.M., Castaño V.M., Gómez-Rodríguez C. (2024). Synthesis and Characterization of SiO_2_ Nanoparticles for Application as Nanoadsorbent to Clean Wastewater. Coatings.

[22] Yuan C., Li Y., Li Q., Jin R., Ren L. (2018). Purification of Tea Saponins and Evaluation of its Effect on Alcohol Dehydrogenase Activity. Open Life Sci..

[23] Agarwal S., Murthy R.S.R., Harikumar S.L., Garg R. (2020). Quality by Design Approach for Development and Characterisation of Solid Lipid Nanoparticles of Quetiapine Fumarate. Curr. Comput. Aided Drug Des..

[24] Paswan S.K., Saini T.R. (2021). Comparative Evaluation of In Vitro Drug Release Methods Employed for Nanoparticle Drug Release Studies. Drug Technol..

[25] Manikandan M., Kannan K. (2016). Study on in vivo Release and in vivo Absorption of Camptothecin-Loaded Polymeric Nanoparticles: Level A in vitro-in vivo Correlation. Asian J. Pharm. Clin. Res..

[26] Ghasemi M., Turnbull T., Sebastian S., Kempson I. (2021). The MTT Assay: Utility, Limitations, Pitfalls, and Interpretation in Bulk and Single-Cell Analysis. Int. J. Mol. Sci..

[27] Rapa S.F., Waltenberger B., Di Paola R., Adesso S., Siracusa R., Peritore A.F., D’Amico R., Autore G., Cuzzocrea S., Stuppner H. (2020). Plumericin prevents intestinal inflammation and oxidative stress in vitro and in vivo. FASEB J..

[28] Hartinger J., Veselý P., Šíma M., Netíková I., Matoušková E., Petruželka L. (2017). 5-Fluorouracil Toxicity Mechanism Determination in Human Keratinocytes: In Vitro Study on HaCaT Cell Line. Prague Med. Rep..

[29] Rapa S.F., Magliocca G., Pepe G., Amodio G., Autore G., Campiglia P., Marzocco S. (2021). Protective Effect of Pomegranate on Oxidative Stress and Inflammatory Response Induced by 5-Fluorouracil in Human Keratinocytes. Antioxidants.

[30] Charachit N., Sukhamwang A., Dejkrajangkraikul P., Yodkeeree S. (2022). Hyperoside and Quercitrin in *Houttuynia cordata* Extract Attenuate UVB-Induced Human Keratinocyte Cell Damage and Oxidative Stress via Modulation of MAPKs and Akt Signaling Pathway. Antioxidants.

[31] Cory G. (2011). Scratch-Wound Assay. Methods Mol. Biol..

[32] Koonyosying P., Paradee N., Srichairatanakool S. (2023). Hormetic Effects of Plant Bioactives on Healthy Aging and Longevity. Plant Bioactives as Natural Panacea Against Age-Induced Diseases.

[33] Curthoys G., Davydov V.Y., Kiselev A.V., Kiselev S.A., Kuznetsov B.V. (1974). Hydrogen Bonding in Adsorption on Silica. J. Colloid Interface Sci..

[34] Nepal B., Bhattarai J.K., Dhami K.B., Nichols M.R., Stine K.J. (2023). Effect of Mesoporous Silica Nanoparticles Loaded with α-Tomatine on HepG2 Cancer Cells Studied In Vitro. J. Drug Deliv. Sci. Technol..

[35] Wang X., Tang H., Wang C., Zhang J., Wu W., Jiang X. (2016). Phenylboronic Acid-Mediated Tumor Targeting of Chitosan Nanoparticles. Theranostics.

[36] Gaonkar R.H., Ganguly S., Dewanjee S., Sinha S., Gupta A., Ganguly S., Chattopadhyay D., Debnath M.C. (2017). Garcinol Loaded Vitamin E TPGS Emulsified PLGA Nanoparticles: Preparation, Physicochemical Characterization, In Vitro and In Vivo Studies. Sci. Rep..

[37] EL-Rafei A.M. (2022). Preparation and Characterization of Mesoporous Amorphous Nano-Silica and Nano-Cristobalite for Value Enhancement of Low-Cost Egyptian Waste Materials. Ceram. Int..

[38] Singh L.P., Bhattacharjya S.K., Shah S.P., Mishra G., Sharma U. (2016). Studies on Early Stage Hydration of Tricalcium Silicate Incorporating Silica Nanoparticles: Part II. Constr. Build. Mater..

[39] Silva N., Correia P.R.C., Druzian J., Fakhouri F.M., Fialho R.L.L., Cabral-Albuquerque E. (2017). PBAT/TPS Composite Films Reinforced with Starch Nanoparticles Produced by Ultrasound. Int. J. Polym. Sci..

[40] Saedi S., Shokri M., Priyadarshi R., Rhim J.W. (2021). Carrageenan-Based Antimicrobial Films Integrated with Sulfur-Coated Iron Oxide Nanoparticles (Fe_3_O_4_@SNP). ACS Appl. Polym. Mater..

[41] Demento S.L., Eisenbarth S.C., Foellmer H.G., Platt C., Caplan M.J., Saltzman W.M., Mellman I., Ledizet M., Fikrig E., Flavell R.A. (2009). Inflammasome-Activating Nanoparticles as Modular Systems for Optimizing Vaccine Efficacy. Vaccine.

[42] Mahony D., Cavallaro A.S., Stahr F., Mahony T.J., Qiao S.Z., Mitter N. (2013). Mesoporous Silica Nanoparticles Act as a Self-Adjuvant for Ovalbumin Model Antigen in Mice. Small.

[43] Chen Y., Tang Y., Li Y., Rui Y., Zhang P. (2024). Enhancing the Efficacy of Active Pharmaceutical Ingredients in Medicinal Plants through Nanoformulations: A Promising Field. Nanomaterials.

[44] Hou Y.-T., Wu K.C.-W., Lee C.-Y. (2019). Development of Glycyrrhizin-Conjugated, Chitosan-Coated, Lysine-Embedded Mesoporous Silica Nanoparticles for Hepatocyte-Targeted Liver Tissue Regeneration. Mater. Sci. Eng. C.

[45] Pasieczna-Patkowska S., Cichy M., Flieger J. (2025). Application of Fourier Transform Infrared (FTIR) Spectroscopy in Characterization of Green Synthesized Nanoparticles. Molecules.

[46] Aulifa D.L., Amarilis B., Ichsani L.N., Maharani D.S. (2024). A Comprehensive Review: Mesoporous Silica Nanoparticles Greatly Improve Pharmacological Effectiveness of Phytoconstituents in Plant Extracts. Pharmaceuticals.

[47] Kerry R.G., Singh K.R.B., Mahari S., Jena A.B., Panigrahi B. (2023). Bioactive Potential of Morin Loaded Mesoporous Silica Nanoparticles: A Noble and Efficient Antioxidant, Antidiabetic, and Biocompatible Agent. OpenNano.

[48] Singh L.P., Bhattacharyya S.K., Ahalawat S. (2015). Polymer Functionalized Silica Nanoparticles: A Comprehensive Review on Surface Properties and Applications. Constr. Build. Mater..

[49] Hu Y., Cui X., Zhang Z., Chen L., Zhang Y., Wang C., Yang X., Qu Y., Xiong Y. (2018). Optimisation of Ethanol-Reflux Extraction of Saponins from Steamed *Panax notoginseng* by Response Surface Methodology and Evaluation of Hematopoiesis Effect. Molecules.

[50] Danaei M., Dehghankhold M., Ataei S., Hasanzadeh Davarani F., Javanmard R., Dokhani A., Khorasani S., Mozafari M.R. (2018). Impact of Particle Size and Polydispersity Index on the Clinical Applications of Lipidic Nanocarrier Systems. Pharmaceutics.

[51] Xiong Y., Dong Z., Zhou H., Mao J., Zeng L., Jiang Y., Meng F., Liao Z., Chen M. (2024). Toxicokinetics and Tissue Distribution of the Hepatotoxic Triterpenoid Saponin Pterocephin A in Rats Using the Ultra-Performance Liquid Chromatography-Tandem Mass Spectrometry (UPLC-MS/MS) Method. Molecules.

[52] Trzeciak K., Chotera-Ouda A., Bak-Sypien I.I., Potrzebowski M.J. (2021). Mesoporous Silica Particles as Drug Delivery Systems—The State of the Art in Loading Methods and the Recent Progress in Analytical Techniques for Monitoring These Processes. Pharmaceutics.

[53] Khan M.I., Karima G., Khan M.Z., Shin J.H., Kim J.-D. (2022). Therapeutic effects of saponins for the prevention and treatment of cancer by ameliorating inflammation and angiogenesis and inducing antioxidant and apoptotic effects in human cells. Int. J. Mol. Sci..

[54] Budiman A., Rusdin A., Wardhana Y.W., Puluhulawa L.E., Mo’o F.R.C., Thomas N., Gazzali A.M., Aulifa D.L. (2024). Exploring the transformative potential of functionalized mesoporous silica in enhancing antioxidant activity: A comprehensive review. Antioxidants.

[55] Qiu T., Gu P., Wusiman A., Ni H., Xu S., Zhang Y., Zhu T., He J., Liu Z., Hu Y. (2021). Immunoenhancement Effects of Chitosan-Modified Ginseng Stem-Leaf Saponins-Encapsulated Cubosomes as an Adjuvant. Colloids Surf. B Biointerfaces.

[56] Dong Y., He Y., Fan D., Wu Z. (2023). Preparation of pH-Sensitive Chitosan-Deoxycholic Acid-Sodium Alginate Nanoparticles Loaded with Ginsenoside Rb_1_ and Its Controlled Release Mechanism. Int. J. Biol. Macromol..

[57] Tsai C.L., Changchien C.Y., Chen Y., Chang H.H., Tsai W.C., Wang Y.W., Chou K.C., Tsai H.C., Wang C.Y., Chiang M.H. (2022). Accelerated Wound Healing and Keratinocyte Proliferation through PI3K/Akt/pS6 and VEGFR2 Signaling by Topical Use of Pleural Fluid. Cells.

[58] Lee J.H., Kim H.L., Lee M.H., You K.E., Kwon B.J., Seo H.J., Park J.C. (2012). Asiaticoside Enhances Normal Human Skin Cell Migration, Attachment, and Growth in Vitro Wound Healing Model. Phytomedicine.

[59] Jangra S., Sharma B., Jangra R., Chhokar V., Duhan S. (2018). Saponin-Loaded SBA-15: Release Properties and Cytotoxicity to Panc-1 Cancer Cells. J. Porous Mater..

[60] Sun K., Yu W., Ji B., Chen C., Yang H., Du Y., Song M., Cai H., Yan F., Su R. (2020). Saikosaponin D Loaded Macrophage Membrane-Biomimetic Nanoparticles Target Angiogenic Signaling for Breast Cancer Therapy. Appl. Mater. Today.

[61] Guo C., Su Y., Wang H., Cao M., Diao N., Liu Z., Chen D., Kong M. (2022). A Novel Saponin Liposomes Based on the Couplet Medicines of *Platycodon grandiflorum*-*Glycyrrhiza uralensis* for Targeting Lung Cancer. Drug Deliv..

[62] Mody K.T., Popat A., Mahony D., Cavallaro A.S., Yu C., Mitter N. (2013). Mesoporous Silica Nanoparticles as Antigen Carriers and Adjuvants for Vaccine Delivery. Nanoscale.

[63] Ling J., Cai Y., Feng H., Liu Z., Ouyang X.-K. (2024). Polydopamine-Modified Copper Coordination Mesoporous Silica Nanoparticles Loaded with Disulfiram for Synergistic Chemo-Photothermal Therapy. Pharmaceutics.

