# Supplementary material for: Developments and Assessments of Crude Tea Saponin-Incorporated Silica Nanoparticles for Their Bioactivity Improvement
Source: J Funct Biomater. 2025 Oct 17;16(10):390. doi: 10.3390/jfb16100390 (PMC12565442; doi:10.3390/jfb16100390)
Supplement: Supplementary file 1 [file jfb-16-00390-s001.zip › jfb-3859082-supplementary.pdf]

**Supplementary Data**

The quantitative results for TS and TSNPs, including their compound concentrations and corresponding saponin contents, are summarized in Table S1.

**Table S1** Quantification of saponin content in TS and TSNPs based on HPLC peak area

| Compound concentration (mg/mL) ($\mathbf{Z_{TS}}$ or $\mathbf{Z_{TSNPs}}$) | TS | | TSNPs | |
|---|---|---|---|---|
| | Main peak area (mAu*s) ($\mathbf{Y_{TS}}$) | Saponin concentration of TS (mg/mL) ($\mathbf{X_{TS}}$) | Main peak area (mAu*s) ($\mathbf{Y_{TSNPs}}$) | Saponin concentration of TSNPs (mg/mL) ($\mathbf{X_{TSNPs}}$) |
| 3.1 | 3229.7 | 1.4 | 2223.2 | 1.4 |
| 6.2 | 6641.0 | 2.8 | 4427.2 | 2.5 |
| 12.5 | 13047.4 | 5.6 | 8752.8 | 4.7 |
| 25.0 | 25198.7 | 11.3 | 17209.2 | 9.0 |
| 50.0 | 46778.9 | 22.5 | 37432.9 | 19.4 |

$\mathbf{X_{TS}}$: Saponin content is quantified based on the main peak in the HPLC chromatogram (retention time approximately 25.62), representing approximately 45% of the total peak area. The calculated saponin concentration is derived from the formula ($X_{TS}$ = total sample concentration($Z_{TS}$) x 0.45).

$\mathbf{X_{TSNPs}}$: The values were calculated using the equation ($Y_{TSNPs} = 1956.9\ X_{TSNPs} - 472.1$) from a concentration curve of TS relative peak area equation, where $X_{TS}$ and $Y_{TS}$ were converted to $X_{TSNPs}$ and $Y_{TSNPs}$, respectively.

The calculated saponin equivalency ($X_S$) in TSNPs relative to TS (Table S2), based on the equation 1 and 2 ;

$$Z_{TS} = 2.2223X_S - 0.0017\ (R^2 = 1) \text{ ---------- (equation-1)}$$

$$Z_{TSNPs} = 2.6087X_S + 0.0569\ (R^2 = 0.9985) \text{ ---------- (equation-2)}$$

**Table S2** Saponin equivalency in TSNPs relative to TS based on calculated values

| Saponin equivalency (mg/mL) ($\mathbf{X_S}$) | TS concentration (mg/mL) ($\mathbf{Z_{TS}}$) | TSNPs concentration (mg/mL) ($\mathbf{Z_{TSNPs}}$) |
|---|---|---|
| 1.4 | 3.1 | 3.7 |
| 2.8 | 6.2 | 7.4 |
| 5.6 | 12.5 | 14.7 |
| 11.3 | 25.0 | 29.4 |
| 22.5 | 50.0 | 58.8 |
